# Controlled administration of cannabis to mitigate cannabis-attributable harm among recreational users: a quasi-experimental study in Germany

**DOI:** 10.12688/f1000research.22612.2

**Published:** 2020-10-06

**Authors:** Jakob Manthey, Jens Kalke, Jürgen Rehm, Moritz Rosenkranz, Uwe Verthein

**Affiliations:** 1 Institute of Clinical Psychology and Psychotherapy, Technische Universität Dresden, Dresden, Germany; 2 Centre for Interdisciplinary Addiction Research, Department of Psychiatry, University Medical Center Hamburg-Eppendorf, Hamburg, Germany; 3 Institute for Mental Health Policy Research and Campbell Family Mental Health Institute, Centre for Addiction and Mental Health, Toronto, ON, Canada; 4 Dalla Lana School of Public Health and Department of Psychiatry, University of Toronto, Toronto, ON, Canada; 5 Department of International Health Projects, Institute for Leadership and Health Management, I.M. Sechenov First Moscow State Medical University, Moscow, Russian Federation

**Keywords:** Cannabis, Marihuana, THC, legal, prohibition, Germany, model study, pharmacy

## Abstract

**Background: **New approaches are required to slow down or reverse increasing trends of levels of delta-9-tetrahydrocannabinol (THC) and cannabis-attributable hospitalizations in Germany. Legal access to cannabis may constitute one viable effective policy response; however, available evidence does not suffice to inform a regulation model for Germany. The proposed study aims to reduce harm for cannabis users through legal access to herbal cannabis through pharmacies.

**Protocol:** A quasi-experimental study comparing cannabis users with legal access to herbal cannabis (Berlin, intervention group) to those without legal access (Hamburg, control group) (total N=698). As the primary outcome, we hypothesize that: 1) illegal THC consumption will reduce by at least 50% in the intervention group and 2) total THC exposure in the intervention group will be reduced by at least 10% lower than that of the control group, taking into account baseline values. Secondary outcomes comprise measures of frequency of use, THC-impaired driving, and mode of administration. Paired t-tests and multilevel regression models will be performed for statistical analyses.

**Discussion: **This study proposal is currently being reviewed by the ‘Federal Institute for Drugs and Medical Devices’ – the body responsible for approving research studies on classified substances, including cannabis. Upon approval and prior to the start of the study, a full ethical review will be undertaken. Results may inform a regulation model for Germany and other jurisdictions and are expected to deepen the understanding of the effects of legal access to cannabis.

**Pre-registration: **German Clinical Trials Register (DRKS), DRKS00020829

## Introduction

Cannabis remains the most prevalent illegal drug in Europe and other regions worldwide ^1^. In the EU, 14.4% of people aged 15–34 years indicated past-year use of cannabis ^2^. In the past decade, several trends adverse to public health have been observed, including rising prevalence of cannabis use, growing treatment demand for cannabis problems ^3^, and increases in potency levels in both herbal cannabis and resin ^4^. These trends are also mirrored in Germany, where, since 2006, prevalence of past-year use increased by 75% ^5^, treatment demand increased by 113%, and potency of resin increased by 119% (data for herbal cannabis not available ^6^). Moreover, the number of cannabis-related offences has risen by 38% in the same period ^7^.

To date, effective policy responses to the above-outlined trends could not be observed. In fact, these trends demonstrate that current cannabis policies in Germany have not resulted in reductions in drug demand. Furthermore, the possibilities for reducing or slowing down increases in potency are very limited in an illegal or unregulated drug market. However, potency levels constitute a crucial determinant for public health as the main ingredient, delta-9-tetrahydrocannabinol (THC), has been linked to severity of cannabis dependence ^8^, cognitive impairment ^9^, and incidence of psychotic disorders ^10^. Consequently, THC concentrations in legally available products are deemed to be increasingly important by policymakers ^11, 12^. With the legalization of recreational cannabis in Canada and several US American states, it has become mandatory to label all commercially produced cannabis products with their respective THC level (for Canada, see 13, 14).

In addition to the lack of control over potency levels, illegal markets also make it impossible to establish minimum criteria for safety and purity of available products. Analyses of herbal cannabis acquired on the Swiss black market showed that only one in three samples passed the microbiological test for human consumption ^15^. According to one systematic review, microbes, heavy metals, and pesticides constitute the most prevalent contaminants in cannabis products ^16^. While the risks from contaminants to human health have not yet been quantified, case reports highlight that the use of contaminated products may be potentially life-threatening ^17^.

Consequently, legalizing the cannabis market would allow for the control over the rising THC exposure and cannabis-attributable sequelae, and to ensure safety standards of available products are being met. However, there is a multitude of options to realize legal access to cannabis. One option is the creation of so-called ‘cannabis social clubs’, which are established by consumers with the aim of growing and distributing herbal cannabis on a non-for profit basis (e.g., in Uruguay, Spain, Belgium ^18, 19^). More prominently, legal markets in North America have been established by private retailers or government monopolies, while some jurisdictions allow users to grow their own cannabis themselves.

A combination of different modes of access to legal cannabis can be observed in Uruguay, where users may grow their own cannabis, acquire cannabis through membership in a social club or through licensed pharmacies, which get a limited supply of herbal cannabis from government-licensed suppliers ^20, 21^. In fact, a pilot project was proposed in 1997 for a regulated sale of cannabis in pharmacies in a German state ^22^. While this proposal was never implemented due to lack of political support, the ‘pharmacy model’ has several advantages over other private models as pharmacists are trained to test substances with regard to purity and are also more familiar with recognizing substance misuse than are commercial vendors, especially those who are selling product over the Internet. Further, pharmacists are already familiar with dispensing medical marijuana in Germany, and already meet the requirements for dealing with classified substances, such as cannabis.

To inform a cannabis regulation model for Germany, evidence gathered from evaluations of jurisdictions legalizing cannabis in the Americas may serve as a useful base. However, North American cannabis users differ from European users in regard to use modes (e.g. lower co-use of tobacco ^23^, higher use of concentrates ^24, 25^) and patterns (e.g., the stark increase of daily use in the USA ^26^), demanding different requirements for a regulation model in Germany. Further, evidence collected from large-scale natural experiments has its limitations, which may be overcome in small-scale, controlled experiments ^27^. Thus, evaluating tightly regulated cannabis administration models in European jurisdictions is required.

In this study protocol, we outline the proposal for the regulated sale of cannabis to a limited number of users in Berlin, Germany. Responding to calls to make cannabis safer ^28^, this study sets out to reduce harm to recreational users through: a) use of legal cannabis products free from contaminants and pollutants, and b) capping maximum THC-levels and incentivizing the purchase of low-potency cannabis products by aligning retail prices with potency levels and thereby reducing THC exposure among users.

## Protocol

Version: 1 (26 February 2020).

This protocol has been written in accordance with the SPIRIT guidelines ^29^.

### Study design, sample and recruitment

**Figure 1.**
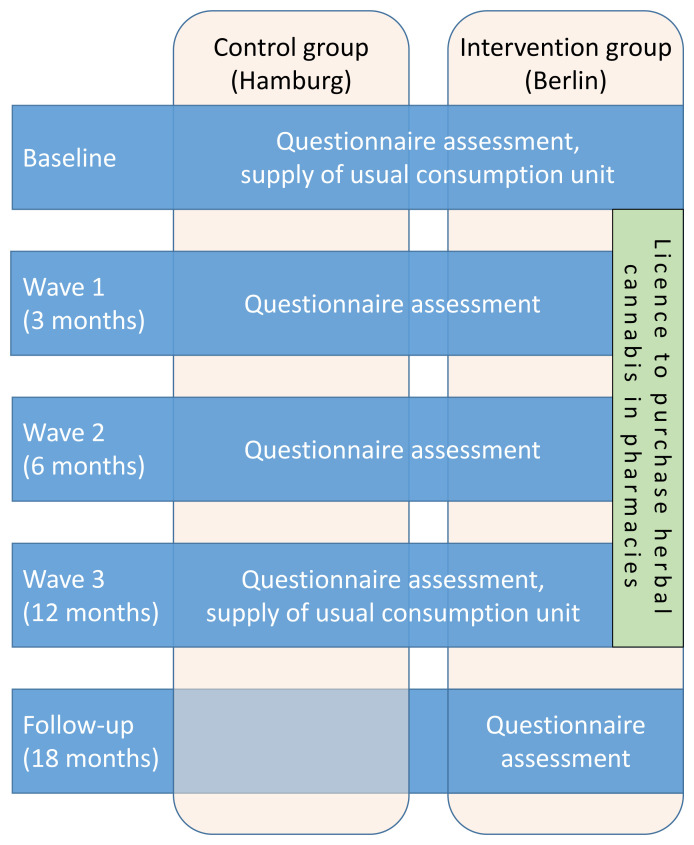
Study flow chart.

The aims of the study will be evaluated using a quasi-experimental two-group study design, as summarized in Figure 1. The intervention will constitute an individualized licence to purchase herbal cannabis in selected pharmacies for a duration of 12 months in Berlin, Germany. Control group participants will be recruited in Hamburg, a city comparable to Berlin in terms of sociodemographic indicators (population, deprivation) and user characteristics ^30^.

Prior to any data collection, study team members will take consent of each participant. A model consent form is available as *Extended data* ^31^. In both study groups, participants will be asked to complete a questionnaire and provide their usual consumption unit (e.g., joints, mix of tobacco and cannabis to smoke in water pipe, etc.) at both baseline and again after one year (T12). In predetermined intervals, additional questionnaires will be completed by all participants. Further, individual sales data will be collected for intervention group participants, which will be part of the primary outcome analyses.

All participants will be recruited via posters, or through consumer groups and local stakeholders in each city. To incentivize study participation and minimize loss to follow-up, participants will be reimbursed with 25€ upon completion of the questionnaires at each wave. Further, providing details regarding their usual consumption unit will be reimbursed with 15€ (at baseline and at one-year follow-up).

Every resident in the respective control or intervention municipality aged 18 years or above reporting to have used cannabis at least monthly in the past 6 months may participate in the study. Following international recommendations to minimize cannabis use risks ^32^, persons with a familial predisposition to psychotic or substance use disorders, pregnant women, persons in current psychiatric treatment or with a preceding psychotic disorder will be excluded from this study. Further, persons with a prescription to use cannabis for medical purposes will not be eligible to participate as the study focuses on recreational use only.

### Details of the dispensary model

The term dispensary model describes how intervention group participants can acquire cannabis in pharmacies and all measures undertaken to achieve the postulated aim of mitigating use risks. Registered users will be eligible to purchase up to 5 grams of herbal cannabis per transaction and up to 15 grams of herbal cannabis per week. All herbal cannabis will be sold in 1-gram packages, which will be labelled with the concentration of THC and Cannabidiol (CBD, ^33, 34^), and contain further information (e.g., on other constituents, producer, date of production, safety warnings).

Two core measures will deter intervention group participants from using high-potency cannabis varieties. First, we will restrict the sale to varieties containing at most 12% THC, which is just below the median value of 13% identified in herbal cannabis batches seized by the federal police in 2017 ^35^. Second, the retail price will be bound to the THC level, with lower potencies being more affordable. Specifically, the variety lowest in THC will be sold at the current black-market price, as determined by the federal police. More potent varieties will be sold at higher prices, with the most potent variety to be sold at 50% above the current black-market price. In this way, purchasing varieties with high THC levels will be disincentivized.

To further mitigate the risks of THC, an upper threshold of the THC/CBD ratio will be established as CBD has been found to attenuate some of the adverse effects of THC (see e.g. ^9, 36^). In the absence of any systematic evidence on the specific risk profile of THC/CBD ratios, an upper threshold of 50:1 was defined based on US data, indicating a worrying ratio increase from 23:1 to 104:1 between 2008 and 2017 ^25^.

All herbal cannabis to be sold in the pharmacies will be acquired through the medical supply system ^37^. As of 2019, six out of the 20 varieties available for medical use in Germany ^38^ met the outlined criteria (cap of potency and cap of THC/CBD ratio) to be sold to the intervention group participants in this study. As prescribed for medical cannabis, each batch of herbal cannabis will undergo routine pharmaceutical tests. Further, producers of medical marijuana are legally required to perform systematic tests to detect variations in potency levels and contaminants. In accordance with the requirements of the narcotics law, cannabis stored in the pharmacies will not be accessible to anyone but trained staff. All cannabis products will be stored in a steel cabinet protected by a safety lock, in accordance with the standards prescribed by the German ‘Federal Institute for Drugs and Medical Devices’ (Bundesinstitut für Arzneimittel und Medizinprodukte, BfArM).

In all participating pharmacies, users will find printed information material on mitigating use risks and will be offered the option of renting devices for vaporizing herbal cannabis. The use of vaporizers can reduce exposure to some hazardous compounds and is hence considered a harm-reducing method ^39^. Further, the vending staff in all participating pharmacies will be trained in the following: legal framework, safer use guidelines (original: ^32^, for a German translation: ^40^), identification of vulnerable users, cooperation with drug counselling and prevention services. If required, study participants will be able to hold conversations with staff in private on the pharmacy premises.

### Primary outcome

Two important aspects will determine the study’s success and will be combined in the primary outcome, measured at baseline and at 12-month follow-up: 1) dominance of legal over illegal consumption, and 2) reduction of THC exposure. The hypothesis for the primary outcome is:

“As compared to baseline, intervention group participants will reduce their use of illegally acquired THC within the past 30 days by at least 50% at 12-month follow-up. Taking into account baseline exposure levels, the total amount of THC consumed among *intervention* group participants within the past 30 days will fall below the total amount of THC consumed among *control* group participants within the past 30 days by at least 10% at 12-month follow-up.”

As first aspect, we expect that illicit cannabis use will be largely replaced by consumption of legally acquired products. A shift from an illegal to a legal consumption environment implies that exposure to contaminants and pollutants but also legal consequences (revocation of driver’s licence, imprisonment) will be largely diminished or even completely prevented. As an evaluation of changes in these specific outcomes would require very large sample sizes, and go beyond the scope of this study, we will restrict the first part of the primary outcome to a proxy, i.e., to the percentage of legal cannabis consumption. This will be operationalized as the percentage of an individual’s THC exposure within the past 30 days acquired through purchases in pharmacies.

For the second aspect, we expect to halt the increasing THC exposure among intervention group users. While THC levels in seized and legally available cannabis products are increasing, intervention group participants are expected to reduce their overall THC consumption as compared to control group participants. While THC exposure is the core determinant for cannabis-attributable harm, specific effects on adverse consequences (e.g. on incidence of psychotic disorders) require larger sample sizes which, again, are beyond the scope of this study.

While the first aspect will only be evaluated within the intervention group, the second aspect will require between-group comparisons across a 12-month period. Only if both aspects can be positively evaluated, can an overall success of the study be inferred.

### Secondary outcomes

For secondary outcomes, we will adhere to the criteria proposed to evaluate the legalization of cannabis in Canada ^41^, encompassing all cannabis use behaviours linked to adverse consequences, for which substantial evidence exists ^32^. As intervention group participants will receive information on risky cannabis use ^32^ and enclosed within each cannabis package sold by the pharmacies, we hypothesize that risky cannabis use practices will not increase among these users – despite the presumably increased availability of cannabis products. Specifically, we hypothesize that intervention group participants will: A) not increase their use frequency, B) not increase frequency of THC-impaired driving, and C) will not smoke cannabis products more frequently (for operationalization, see Table 1). The secondary outcomes will be evaluated at one-year follow-up, which involves testing for differences between control and intervention group while taking baseline values into account.

**Table 1.** Operationalization of secondary outcomes.

Indicator	Operationalization
A) Frequency of use	Number of use days in the past 30 days
B) THC-impaired driving	Number of occasions on which a vehicle was driven within six hours after using cannabis in the past 30 days
C) Mode of administration	Percentage of all use units using combustible methods, such as smoking joints, blunts, (water) pipes

As further secondary outcomes, we will examine acceptance and satisfaction of the dispensary model among intervention group participants, which may be important for explaining a possible dominance of illegal over legal consumption (see first aspect of primary outcome).

### Assessment details

For both aspects of the primary outcome, THC exposure within the past 30 days needs to be determined for each respondent by combining the following data sources. First, THC levels per average use occasion will be determined through analyses of usual consumption samples provided at both baseline and after one year (T12). Second, questionnaire data will be used to determine the number of use occasions within the past 30 days, separately for legally and illegally acquired cannabis. Third, and only for the intervention group, sales data will be used to determine THC exposure levels of legally acquired cannabis over a 12-month period.

In summary, for the control group, THC exposure levels within the past 30 days will be determined multiplying THC levels in a usual consumption unit with the number of use occasions reported in the questionnaire. For the intervention group, THC exposure levels will be determined analogously while correcting for the proportion of legally acquired and consumed THC using sales and questionnaire data.

At all waves — T0, T3, T6, and T12 — a set of questionnaires will be administered (see Table 2). For the primary and secondary outcomes, use characteristics will be assessed using items from the ‘Daily Sessions, Frequency, Age of Onset, and Quantity of Cannabis Use Inventory’ (DFAQ-CU), which will be translated and adapted for this study. All remaining questionnaire data will serve to explain unexpected findings, to control for potential confounders in the statistical analyses, or for the economic evaluation of this study.

**Table 2.** Summary of questionnaire assessment.

Indicator	Questionnaire or item source
Cannabis use characteristics, for primary and secondary outcomes	Items from the DFAQ-CU ^45^
Sociodemographics	Items taken from the WHO Disability Assessment Schedule (WHODAS-II) ^46^
Social situation and participation	Custom-made items
Clinically relevant psychological symptoms	Brief Symptom Inventory (BSI-18) ^47^
Quality of life	EQ5D ^48^
Use of tobacco, alcohol, and other drugs	Custom-made items, AUDIT-C ^49^, use items from EuropASI ^50^
Risky cannabis use/possible cannabis use disorder	CUDIT-R ^51^
Chronic diseases and their treatment	Custom-made items
Utilization of addiction services (counselling, prevention, therapy), for economic evaluation	Custom-made items
Satisfaction and acceptance of the dispensary model	Custom-made items
Adverse events related to using cannabis	Custom-made items

At T18, we will conduct a post-intervention assessment of intervention group participants to examine how their cannabis use has developed after being denied further legal purchases of cannabis products. This assessment will include all instruments outlined in Table 2, in addition to several free-text items.

There will be three main types of study data: (1) chemical analyses of standard consumption units, (2) sales data, (3) survey responses of participants. For (1), we will adhere to the standard operating procedures issued by the BfArM ^42^. For (3), we will aim to carry out digital survey assessments to minimize human error in data entry. Further, consistency checks will be performed before data analyses.

### Sample size and data analyses

The study outcomes will be analysed according to ‘Intention-to-treat’ (ITT) principles. Specifically, the sample to be analysed is defined as all users who have provided a usual consumption unit within 4 weeks after completing the baseline questionnaire. Over-recruitment will compensate for participants failing to provide a consumption unit within this period. According to ITT principles, only those participants who provided their baseline data, including their usual consumption unit, will be included in the analyses.

To examine between-group differences with a t-test, the required sample size of this study was calculated assuming a power of 80%, a 5% alpha error, and a THC standard deviation equal to one-third of its mean (approximated using Canadian data, ^43^; adding 25% to account for uncertainty). The required sample size was estimated to sum up to n=698 participants (control: n=349; intervention: n=349) for detecting group differences in THC exposure.

Not relevant for sample size considerations, we expect that 20% (control: 30%; intervention: 10%) of all participants of the ITT sample will be lost to T12 follow-up. Subjects dropping out of the study will not be replaced and missing values will be imputed using the ‘Last Observation Carried Forward‘ (LOCF) technique. The critique regarding LOCF imputation methods ^44^ does not apply to our study, as this method will bias the data towards the null hypothesis (assuming no change over time) and therefore representing a conservative imputation approach. The assumptions of LOCF will be examined in sensitivity analyses using advanced multiple imputation techniques.

To evaluate both aspects of the primary outcome, two analyses need to be conducted. First, the THC exposure levels in the past 30 days ascribed to illegal cannabis acquisition within the intervention group needs to be compared between baseline and intervention, with a 50% reduction denoting the minimum threshold for a positive evaluation. Second, a between-group comparison of THC exposure in the past 30 days at T12 adjusting for baseline data will be conducted using a t-test. The dependent variable will be calculated as follows:

$$THC_{T12–adjusted}=\frac{THC_{T12}*100}{THC_{T0}}$$

The actual between-group comparison will be evaluated against the following condition:

$$THC_{T12–adjusted–interventiongroup}<=0.9*THC_{T12–adjusted–control\;group}$$

Multilevel regression analyses will be performed additionally for evaluating the primary outcome, which serve to rule out the impact of possibly confounding variables. For evaluating the secondary outcomes, the proposed analyses (correction for baseline values, comparison via t-test, confirmation via multilevel regression analyses) will be performed analogously.

### Adverse events and stoppage

If more than 60% of the intervention group participants drop out within the first three months, the study will be stopped immediately, because it will be taken as an indicator for subjects not accepting the administration model.

In addition, the Data Safety Monitoring Board (DSMB) will be formed by pharmacists and social workers working in the addiction and youth protection field. As stipulated in Good Clinical Practice guidelines, the DSMB, as an independent and multidisciplinary group will be established to review, at intervals, accumulating trial data, in order to monitor the progress of the trial and to make recommendations on whether to continue, modify or stop the trial for safety or ethical reasons.

Adverse events will be closely monitored and documented. Criteria have been pre-specified to define a preterm stop to the study. Further, the study team will be in close contact with the participating pharmacies in order to capture all events not foreseen at study inception. In regular meetings, the DSMB will evaluate the progress of the study and may decide to stop the study.

### Economic evaluation

In a ‘cost-benefit-analysis’ (CBA), the proposed dispensary model will undergo an additional economic evaluation, in which the economic benefits from attenuated THC exposure will be contrasted to the programme costs ^52^, with estimates applied to Germany as a whole. Building on the approach of a previous CBAs for Australia ^53^ and using the extended framework of generalized cost-effectiveness analyses ^54^, we will compare the following scenarios:

- Null scenario: no implementation of cannabis-specific measures (i.e., no police enforcement, no treatment of cannabis use disorders)
- Status quo: implementation of random traffic controls to reduce THC-impaired driving and psychosocial interventions for cannabis use disorders
- Dispensary model: as ‘status quo’ but with legal sale of herbal cannabis in pharmacies

For the CBA, the so-called net social benefit will be calculated as the difference between projected costs and benefits discounted over the study period. On the cost side, we will consider all economic costs that can be ascribed to cannabis-related law enforcement, treatment of cannabis use disorders and cannabis-attributable diseases (e.g. psychoses), loss of productivity, and all costs pertaining to establishing and maintaining the dispensary model. On the benefit side, we will consider all economic benefits arising in the following domains: reductions in law enforcement and health-care costs, as well as increases in productivity. Based on the CBA results, a cost-utility-analyses will additionally be conducted by estimating the costs required to avoid one ‘disability adjusted life year’ (DALY ^55^) for the scenario of a nationwide implementation of the dispensary model.

### Data collection and dissemination

Data collection will be completed using electronic means and all required measures will be implemented to protect the data and anonymity of all study participants at all times. In particular, the study will adhere to the European ‘General Data Protection Regulation’ (GDPR) according to which, health care data is particularly sensitive and should be handled with the greatest care. Further, cannabis possession will remain a federal crime, further emphasizing the sensitive nature of the study data. Thus, all data will be stored on hard drives encrypted using state-of-the-art encryption techniques (AES-256). Upon completion of the study, the study data will be kept on encrypted hard drives in physically locked cabinets.

Conditioned on the approval from the public study sponsor, all study data shall be made available to other researchers. All efforts will be undertaken to anonymize the study data (in the sense of GDPR) in order to publish the data in public repositories making the data findable, openly accessible, interoperable, and re-usable (FAIR principles issued by the European Union). Upon publication of the primary outcome analyses, study data shall be published alongside the respective code of the statistical programme. Through these means, we hope that our analyses will be reproducible and that the data will be used for other purposes than those described, increasing the merit of this study. Lastly, study results will be published under an open access licence to allow for a widespread recognition of our findings.

### Ethical considerations

This study has not undergone ethical review yet. A full-length study proposal is currently being evaluated by the BfArM. According to the German narcotics law, studies on cannabis may, by an exception, be allowed for scientific purposes and only if approved by the BfArM. Only once a positive decision is received from the BfArM will a complete study outline be reviewed by the responsible ethics board. The study has been pre-registered with the German Clinical Trials Register (a primary registry within the WHO registry network) and will be formally registered upon ethical approval (registry number: DRKS00020829). If any amendments of the outlined study design or protocol will be requested by the BfArM or ethics board, they will be reflected in this publication, as well.

### Limitations

There are several limitations of this study. First, given a lack of randomization, causal inferences cannot be drawn from the study findings without controlling for relevant confounders. We have sought to include a broad variety of questionnaires to capture information on all possible determinants; however, we cannot rule out that some important confounding variables will not be assessed or be biased through self-report. Further, resin will not be part of the dispensary model for it is not available from medical suppliers. As far as we know, resin cannot be prescribed for medical purposes despite favourable THC/CBD ratios (for Dutch data, see 56). For cannabis users preferring resin over herbal cannabis, study participation may therefore be unattractive, and this may bias the study sample towards predominant herbal cannabis users.

## Study status

The full-length study proposal was submitted in December 2019 to the BfArM and a response is due in March 2020.

## Conclusion

After decades of prohibition and the prospective rescheduling of cannabis in international treaties ^57^, opportunities to reform the regulation of cannabis will continue to emerge in many countries. As with medical marijuana, there is considerable economic pressure for a liberal market of recreational cannabis ^58^, however, public health concerns should be considered in the decisions to change the regulation for the better ^59^. So far, jurisdictions legalizing cannabis for recreational purposes have done so before studying the effects of these policy changes in a closed environment. While these large-scale natural experiments provide valuable insights, small-scale experiments have the advantage of allowing for the study of the effects on an individual level with more control over confounding variables.

The proposed study covers a comprehensive evaluation of a tightly regulated dispensary model of cannabis for recreational users in Germany. To the knowledge of the authors, it will be the first controlled study to investigate the effects of legal access to cannabis in a spatially and temporarily limited framework. Study findings are expected to shape the discussion on the best regulation model for Germany, for Europe, and globally. The evaluation focusing on the primary psychoactive constituent THC acknowledges the causal pathways of cannabis-attributable harm. Findings are expected to inform policy responses to counteract an increase of THC exposure as observed in many European and North American jurisdictions. For Germany specifically, studies evaluating different modes of access to illicit substances have had considerable impact on legislation in the past ^60, 61^. Thus, this study may accelerate the process of legalizing cannabis in Germany and elsewhere. Lastly, results from the economic evaluation will be of interest to policymakers and will serve as essential argument for regulated models of cannabis legalization.

## Data availability

### Underlying data

No data are associated with this article.

### Extended data

Figshare: Model consent form, https://doi.org/10.6084/m9.figshare.11903301.v1 ^31^

### Reporting guidelines

Figshare: SPIRIT checklist, https://doi.org/10.6084/m9.figshare.11903322.v1 ^29^

Data are available under the terms of the Creative Commons Attribution 4.0 International license (CC-BY 4.0).
