# Supplementary material for: Controlled administration of cannabis to mitigate cannabis-attributable harm among recreational users: a quasi-experimental study in Germany
Source: F1000Res. 2020 Oct 6;9:201. Originally published 2020 Mar 23. [Version 2] doi: 10.12688/f1000research.22612.2 (PMC7400698; doi:10.12688/f1000research.22612.2)
Supplement: Peer review history

# Peer review: Reviewer response for version 2

**DOI:** 10.5256/f1000research.29088.r72547

**Published:** 2020-10-16

**Authors:** Josefine Atzendorf

**Affiliations:** 1 Munich Center for the Economics of Aging (MEA), Max Planck Institute for Social Law and Social Policy, Munich, Germany

I recommend the article for indexing.

Is the study design appropriate for the research question?

Yes

Is the rationale for, and objectives of, the study clearly described?

Yes

Are sufficient details of the methods provided to allow replication by others?

Partly

Are the datasets clearly presented in a useable and accessible format?

Not applicable

Reviewer Expertise:

lifestyle risk factors, mental health, substance use, epidemiology

I confirm that I have read this submission and believe that I have an appropriate level of expertise to confirm that it is of an acceptable scientific standard.

# Peer review: Reviewer response for version 1

**DOI:** 10.5256/f1000research.24962.r67190

**Published:** 2020-08-03

**Authors:** Josefine Atzendorf

**Affiliations:** 1 Munich Center for the Economics of Aging (MEA), Max Planck Institute for Social Law and Social Policy, Munich, Germany

The study protocol describes a very interesting study in order to investigate the effects of legal access to cannabis using a quasi-experimental design. The authors describe the study design, sample, data assessment statistical analyses, data protection issues and the importance of their research object very clearly and detailed.

**Minor remark:**

- The study protocol states: “THC levels per average use occasion will be determined through analyses of usual consumption samples provided at both baseline and after one year (T12). Second, questionnaire data will be used to determine the number of use occasions within the past 30 days. Third, and only for the intervention group, sales data will be used to determine THC exposure levels of legally acquired cannabis over a 12-month period.” (S. 5f). The study protocol does not mention if study participants in the intervention group are being asked about their occasions buying cannabis legally and illegally in the questionnaire. I think it would be interesting to have the number of both occasions since the THC exposure of THC bought illegally might be different.

Is the study design appropriate for the research question?

Yes

Is the rationale for, and objectives of, the study clearly described?

Yes

Are sufficient details of the methods provided to allow replication by others?

Partly

Are the datasets clearly presented in a useable and accessible format?

Not applicable

Reviewer Expertise:

lifestyle risk factors, mental health, substance use, epidemiology

I confirm that I have read this submission and believe that I have an appropriate level of expertise to confirm that it is of an acceptable scientific standard.

# Response

**Published:** 2020-08-24

**Authors:** Jakob Manthey

**Affiliations:** TU Dresden, Germany

In order to facilitate understanding of the assessment of the primary outcome (THC exposure), we have added the following half-sentence in the respective section:

Second, questionnaire data will be used to determine the number of use occasions within the past 30 days **, separately for legally and illegally acquired cannabis**.

THC exposure will thus be quantified for illegally and legally acquired cannabis for the intervention group as this is a prerequisite for analysing the primary outcome.

# Peer review: Reviewer response for version 1

**DOI:** 10.5256/f1000research.24962.r61804

**Published:** 2020-04-02

**Authors:** Alfred Uhl

**Affiliations:** 1 Sigmund Freud Private University (SFU), Vienna, Austria; 2 Addiction Competence Centre of the Austrian Public Health Institute (GÖG), Vienna, Austria

The motivation for the treatment group is to get "legal" cannabis. This is very different from the control situation, where subjects only are awarded 25 Euro for filling in a questionnaire. The protocol does not explain exactly which control variables are used to compensate for this possible bias.

 One idea of the study is to sell cannabis with a limited THC/CBD ratio to the experimental group and to see if the total THC consumption can be reduced that way. This requires knowing the amount and purity of illegally consumed cannabis in both groups though. The amount of cannabis can be measured (provided the subjects give correct answers) - but there is no plan to systematically assess the THC content of the illegally acquired cannabis consumed by both groups, as far as the protocol states.

Is the study design appropriate for the research question?

Partly

Is the rationale for, and objectives of, the study clearly described?

Yes

Are sufficient details of the methods provided to allow replication by others?

Yes

Are the datasets clearly presented in a useable and accessible format?

Not applicable

Reviewer Expertise:

Research methodology, epidemiology, substance use

I confirm that I have read this submission and believe that I have an appropriate level of expertise to confirm that it is of an acceptable scientific standard.

# Response

**Published:** 2020-08-24

**Authors:** Jakob Manthey

**Affiliations:** TU Dresden, Germany

Regarding the different incentives for the two groups of study participants:

 We acknowledge that differing incentives in the two groups may have an impact on their motivation. However, we belief that the vast majority of study participants agreeing to participate in the study, will do so because of their internal motivation in supporting this research. The 25€ compensation will only serve to ensure high rates of follow-up. For the control group, we decided not to provide any compensation, as this could be perceived as incentive to buy (and use) cannabis legally. Prior to conducting primary and secondary outcome analyses, we will compare user characteristics (socio-economic variables and cannabis use indicators) of both groups and will take them into account in all analyses, if applicable.

 Regarding the assessment of THC exposure:

 We have added some details on the assessment of the primary outcome to the text. In both groups, we will collect usual consumption units to determine the THC level per use occasion for baseline and 12 months follow-up. For the intervention group, the THC exposure at follow-up will be determined by triangulating baseline data (i.e. from illegally acquired cannabis), sales data, as well as questionnaire data. This will allow to calculate the proportion of total THC consumption attributable to illegally acquired cannabis and thus lay the foundation for the primary outcome analyses.
